# Repositioning drug strategy against *Trypanosoma cruzi*: lessons learned from HIV aspartyl peptidase inhibitors

**DOI:** 10.1590/0074-02760210386

**Published:** 2022-03-16

**Authors:** Leandro Stefano Sangenito, Claudia Masini d’Avila-Levy, Marta Helena Branquinha, André Luis Souza dos Santos

**Affiliations:** 1 Universidade Federal do Rio de Janeiro, Instituto de Microbiologia Paulo de Góes, Departamento de Microbiologia Geral, Laboratório de Estudos Avançados de Microrganismos Emergentes e Resistentes, Rio de Janeiro, RJ, Brasil; 2 Fundação Oswaldo Cruz-Fiocruz, Instituto Oswaldo Cruz, Laboratório de Estudos Integrados em Protozoologia, Rio de Janeiro, RJ, Brasil; 3 Universidade Federal do Rio de Janeiro, Instituto de Química, Programa de Pós-Graduação em Bioquímica, Rio de Janeiro, RJ, Brasil

**Keywords:** Trypanosoma cruzi, repurposing drugs, HIV peptidase inhibitors, chemotherapy

## Abstract

Chagas disease (CD) is an old neglected problem that affects more than 6 million people through 21 endemic countries in Latin America. Despite being responsible for more than 12 thousand deaths per year, the disease disposes basically of two drugs for its treatment, the nitroimidazole benznidazole and the nitrofuran nifurtimox. However, these drugs have innumerous limitations that greatly reduce the chances of cure. In Brazil, for example, only benznidazole is available to treat CD patients. Therefore, some proof-of-concept phase II clinical trials focused on improving the current treatment with benznidazole, also comparing it with repositioned drugs or combining them. Indeed, repositioning already marketed drugs in view of combating neglected tropical diseases is a very interesting approach in the context of decreased time for approval, better treatment options and low cost for development and implementation. After the introduction of human immunodeficiency virus aspartyl peptidase inhibitors (HIV-PIs) in the treatment of acquired immune deficiency syndrome (AIDS), the prevalence and incidence of parasitic, fungal and bacterial co-infections suffered a marked reduction, making these HIV-PIs attractive for drug repositioning. In this line, the present perspective presents the promising and beneficial data concerning the effects of HIV-PIs on the clinically relevant forms of *Trypanosoma cruzi* (i.e., trypomastigotes and amastigotes) and also highlights the ultrastructural and physiological targets for the HIV-PIs on this parasite. Therefore, we raise the possibility that HIV-PIs could be considered as alternative treatment options in the struggle against CD.

The old neglected problem: Chagas disease

Chagas disease (CD) remains as one of the 20 neglected tropical diseases listed by the World Health Organization (WHO).^1^ Its etiologic agent, the trypanosomatid protozoan *Trypanosoma cruzi*, infects about 6 to 8 million people, resulting in more than 12 thousand deaths every year and leading to severe digestive and heart system problems.^2^ This illness is endemic in 21 countries of the Latin America where Argentina, Bolivia, Mexico and Brazil are in the top in terms of prevalence rates, accounting for approximately 70% (4.2 million) of the estimated cases.^3^ ^,^ ^4^ These numbers are probably underestimated, since it is suggested that less than 10% of the people affected by CD in the world are properly diagnosed.^5^ The Disability-Adjusted Life-Years (DALYs) attributed to CD is about 700,000 DALYs, resulting in an overall economic impact of more than impressive US$ 7.2 billion.^6^ ^,^ ^7^ ^,^ ^8^

Unfortunately, the programs against CD are speculative, long and have high cost, taking into account uncertainties of financial return and discouraging the investments on new drugs by the private sector. Consequently, CD disposes only two drugs developed from empirical selection programs about five decades ago: benznidazole, a nitroimidazole (LAFEPE and Abarax^®^/ELEA), and nifurtimox, a nitrofuran (Lampit^®^/Bayer). Benznidazole has the best efficacy and safety profile compared to nifurtimox, which is generally used as first-line treatment in most endemic countries of Latin America. In Brazil, only benznidazole is approved for clinical use by the Brazilian Health Regulatory Agency (ANVISA - Agência Nacional de Vigilância Sanitária). However, both benznidazole and nifurtimox are not considered satisfactory due to its doubtful efficacy and high toxicity, which cause several side effects due to the necessity of long-term administration, which may require continuous monitoring or premature treatment interruption.^5^ ^,^ ^9^ ^,^ ^10^

The strategy of drug repositioning

The strategy of repositioning/repurposing marketed drugs is interesting in the context of better treatment options, low cost for development and decreased time for approval of new clinical uses. Since the toxicity, pharmacokinetics and safety profiles of these medicines are already well established, they can be evaluated directly in phase II clinical trials.^11^ ^,^ ^12^ In this context, several successful examples have been reported in the field of anti-parasitic action.^13^ For instance, dapsone was repositioned for malaria, while its first indication was for hanseniasis treatment. The arsenal to treat cutaneous and visceral leishmaniasis got the backup of the classical antifungal amphotericin B, the antibacterial paromomycin and the anti-cancer miltefosine. Eflornithine, originally developed as an antineoplasic, was repositioned to treat sleeping sickness (African trypanosomiasis) and may be used in combination with the anti-chagasic nifurtimox.^13^ ^,^ ^14^ ^,^ ^15^ Promising candidates for treating CD explored in proof-of-concept phase II clinical trials include the azoles posaconazole and ravuconazole (and its prodrug fosravuconazole), used primarily to treat fungal infections, and fexinidazole, developed against African trypanosomiasis.^10^

**Repositioning HIV aspartyl peptidase inhibitors for combating *T. cruzi***

*The idea behind the use* - HIV aspartyl peptidase inhibitors (HIV-PIs) were released in the mid-1990s, representing an important turnaround in the fight of HIV-positive individuals worldwide against the acquired immunodeficiency syndrome (AIDS).^16^ The HIV-PIs act on the retrovirus aspartyl peptidase, which is fundamental for the correct replication, protein processing and maturation of the viral particles.^17^ The treatment of HIV-positive people is performed with one of these PIs in combination with at least two other antiretroviral drugs in the so-called highly active antiretroviral therapy (HAART). As a consequence of the viral load reduction due to the HAART regiment, there is an improvement of the immune status that culminates in the reduction of morbidity and mortality related to AIDS, promoting living longer and improved quality of life to the patients.^18^ Indeed, there was a substantial retrenchment in the incidence and mortality due to the opportunistic bacterial, viral, fungal and protozoal co-infections.^19^ ^,^ ^20^ ^,^ ^21^ In this point of view, it also seems clear that HIV patients without HAART are more susceptible to develop leishmaniasis as well as to present clinical and parasitological relapses.^22^

CD reactivation prognosis of HIV/*T. cruzi* co-infected patients is much better during under HAART regimen, with reported survival times as long as five years. Before this era, the prognosis was discouraging, with death occurring in days to few months.^23^ ^,^ ^24^ All these observations above led several groups to evaluate and confirm the effects of HIV-PIs on important opportunistic protozoans such as *Cryptosporidium parvum*, *Toxoplasma gondii*, *Plasmodium* spp. and *Leishmania* spp.^20^ ^,^ ^21^ Therefore, these findings supported the idea of testing these compounds on *T. cruzi*, which the main results will be discussed in the next topics.

*HIV-PIs affect the viability and morphology of T. cruzi trypomastigotes* - The HIV-PIs lopinavir, nelfinavir and ritonavir were highly effective in reducing the viability of infective, culture-derived trypomastigotes of *T. cruzi* (strain Dm28c) after 24 h of treatment in a typically dose-dependent manner. The 50% lethal doses (LD_50_) of nelfinavir and lopinavir were 2.7 and 3.0 μM, respectively, while ritonavir displayed a LD_50_ value of 10.7 μM.^25^ The HIV-PIs were also tested against the Y strain of the parasite. After only 4 h of treatment, six out of the nine tested HIV-PIs (Fig. 1) reduced drastically the trypomastigote viability. LD_50_ values for nelfinavir, lopinavir, saquinavir, ritonavir, indinavir and darunavir were calculated as 8.6, 10.6, 12.3, 18.6, 31.7 and 47.4 μM, respectively. Amprenavir, atazanavir and tipranavir were not responsive against the parasites under the same employed experimental conditions. The reference drug, benznidazole, presented a LD_50_ value (6.9 µM) very similar to that found for the best HIV-PIs, nelfinavir and lopinavir.^26^ The reduced viability was followed by frequent and drastic morphological changes, as follows: ruffling and blebs of the plasma membrane, retraction and torsion of the cell body and loss of flagellum (Fig. 2).^25^ ^,^ ^26^

**Fig. 1:**
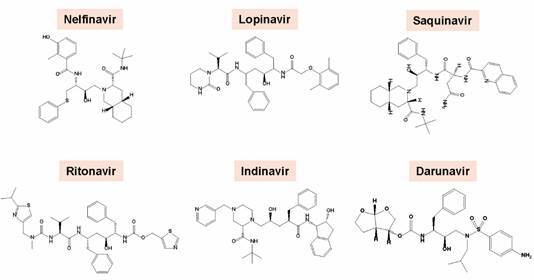
chemical structures of the human immunodeficiency virus aspartyl peptidase inhibitors (HIV-PIs) that presenting relevant anti-*Trypanosoma cruzi* activity.

**Fig. 2:**
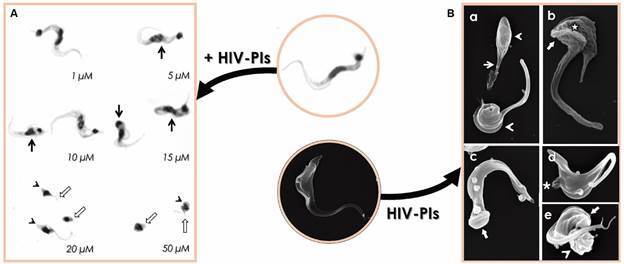
effects of human immunodeficiency virus aspartyl peptidase inhibitors (HIV-PIs) on the morphology and on the ultrastructure of *Trypanosoma cruzi* trypomastigotes Y strain. (Panel A) Giemsa-stained smears of trypomastigotes treated for 4 h with HIV-PIs at concentrations ranging from 1 to 50 µM. The treatment with the HIV-PIs induce numerous morphological changes, such as parasites becoming round in shape with reduced cell size (arrowhead), swollen of cell body (black thin arrow), and shortening or loss of flagellum (white arrow). Note: All HIV-PIs induce such changes. The images are a representative set of the treatment with then. (Panel B) Scanning electron microscopy images of trypomastigotes treated with the LD_50_ dose of nelfinavir (8.6 µM) or lopinavir (10.6 µM) show parasites displaying flagellum shortening (a, thin arrow), body retraction (a and e, arrowhead), ruffling (b, star) and blebs (d, asterisk) of the plasma membrane and atypical body torsion in the posterior end of the cell body (c and e, large arrow). Adapted from Sangenito and coworkers.^26^

*Ultrastructural and physiological targets for HIV-PIs in T. cruzi* - Aspartyl peptidases synthesized by *T. cruzi* cells are hypothesised as promising targets for the HIV-PIs.^27^ ^,^ ^28^ ^,^ ^29^ The enzymatic activity corresponding to the cytoplasmic (soluble fraction) aspartyl-type peptidases was measured in cellular extracts from trypomastigotes of *T. cruzi* Y strain, as judged by the hydrolysis of cathepsin D, a specific fluorogenic peptide substrate. Pepstatin A (10 µM), a prototypal aspartyl PI, blocked the parasite enzyme activity, characterising it as a typical peptidase belonging to this class. The hydrolysis of the substrate was also inhibited by nelfinavir (*ca.* 40%) and lopinavir (*ca.* 50%) at 10 µM.^26^ Indeed, docking binding experiments performed by our group in 2018 revealed that HIV-PIs bind to the active site of an aspartyl peptidase of *T. cruzi*, being lopinavir and ritonavir the ones with greater affinity.^30^ Interestingly, in the same work of Sangenito and colleagues^26^ was reported that the proteasome activity were also inhibited significantly by nelfinavir and lopinavir (at 50 µM) by around 60%. Although this study was the first one to demonstrate the influence of HIV-PIs in the proteasome activity of a pathogenic parasite, since in murine and human models, it has been well-studied that these HIV-PIs can impair the enzymatic activities of 20S and/or 26S proteasomes.^31^ ^,^ ^32^ ^,^ ^33^

In the study of Bellera and co-workers,^34^ the authors used molecular topological descriptors and linear discriminant analysis to obtain a classification model capable of detecting four potential inhibitors of *T. cruzi* cruzipain, the main cysteine peptidase produce by the parasite during different phases along the interaction processes with host structures. Among them, the HIV-PI saquinavir was one of those selected where docking studies predicted interactions between the S2 pocket and the benzyl ring of the PI. However, no strong interaction was detected between the cruzipain catalytic site and saquinavir. Therewith, those authors suggested that the associations with the regions near to the catalytic crevice promoted the stability in the interaction between the complexes.^34^ Corroborating the *in silico* findings, the *in vitro* activity of the partially purified cruzipain was totally blocked by saquinavir at 100 μM.^34^ Similarly, nelfinavir was also capable in reducing the cruzipain-like activity present in the trypomastigotes’ extracts (Y strain). At 10 μM, the HIV-PIs displayed 32% of inhibition, while the classic cysteine PI E-64 reduced the proteolysis of the cruzipain fluorogenic substrate by approximately 43%.^35^

Ultrastructural analyses (Fig. 2) of culture-derived trypomastigotes of *T. cruzi* Y strain revealed that the treatment for 4 h with nelfinavir (at LD_50_ dose of 8.6 µM) and lopinavir (at LD_50_ dose of 10.6 µM) promoted a remarkable shedding of the cytoplasmic membrane, but concentrating mainly in the flagellum of the parasite. The plasma membrane injuries were also confirmed by fluorescence microscopy analysis using propidium iodide as a probe. Both HIV-PIs induced a strong dilation of endoplasmic reticulum with formation of intracellular lipid droplets in near contact to the organelle. Indeed, Nile red staining confirmed the augment of the lipid droplet content throughout parasite cytoplasm.^36^ The treatment also led to a strong swelling of mitochondrion, with rare fragmentation of the organelle matrix. The extent of mitochondrial damage was also accessed by a series of biochemical analyses. Lopinavir and nelfinavir at the LD_50_ doses also reduced the activity of mitochondrial dehydrogenases in 34.3% and 46.8%, respectively. In addition, the incubation with the fluorochrome JC-1 revealed that both compounds at the doses of half of LD_50_ and LD_50_ promoted a high mitochondrial membrane depolarisation, and a nearly complete dissipation of the mitochondrial membrane potential (ΔΨ*m*) with those HIV-PIs at 2×LD_50_ concentration. Both compounds also induced oxidative stress by the highly increased production of reactive oxygen species. Other alterations frequently found by ultrastructural analyses were the appearance of autophagosomes and the strong dilation of the nuclear envelope, but without affecting the nuclear DNA.^36^

The data presented herein clearly evidenced several targets for HIV-PIs in *T. cruzi* parasites. Particularly, nelfinavir and lopinavir target vital cellular structures, such as the plasma membrane, endoplasmic reticulum and the mitochondrion. Alterations in the plasma membrane are usually related on *T. cruzi* under treatment with different drugs due to the osmotic stress unleashed or the attempt of the parasite to promote the shedding of the compound.^37^ ^,^ ^38^ Interestingly, several studies using human and murine cell models demonstrated that HIV-IPs extensively affect the mitochondrion and the endoplasmic reticulum. Misbalance in these organelles are directly associated with lipid disturbances induced by HIV-PIs in HIV-positive patients.^35^ ^,^ ^39^ Therefore, it appears that the effects of the HIV-PIs on *T. cruzi* is very similar to those present in higher eukaryotes. However, further studies need to be conducted to confirm this hypothesis. Some peculiar alterations induced by the HIV-PIs are suggestive of distinct death phenotypes which reinforces the need for better understanding these mechanisms in *T. cruzi*. For example, damages of plasma membrane is attributed to necrotic phenotypes, while the imbalance in the correct mitochondrial functionality is usually observed in classical apoptosis.^40^ Also, the presence of autophagosomes could be indicative of cell death by autophagy.^41^

Finally, nelfinavir and lopinavir also modulated the expression of some surface molecules of trypomastigotes (Y strain) that play important roles in many physiopathological events. The use of peanut agglutinin as a probe, which avidly binds to α-galactose residues of mucin-like glycoproteins, demonstrated that nelfinavir treatment downregulated (*ca.* 22.5%) the expression of these surface molecules. The treatment with both HIV-PIs also reduced the expression of molecules containing sialic acid by 18.5% and 13.9%, respectively, when the *Maackia amurensis* agglutinin was used. These results revealed that both HIV-PIs reduced the ability of parasites to sialylate their mucins, probably by affecting the expression of the mucin coat, the receptor sites or directly acting on *trans*-sialidase activity.^35^ Therefore, these findings open the possibility for further studies.

*HIV-PIs impaired the interaction process with mammalian host cells and impact the viability of the intracellular replicative amastigotes of T. cruzi* - The impact of HIV-PIs was also accessed on the (1) interaction process of *T. cruzi* trypomastigotes (Y strain) with host cells and (2) development and replication of intracellular amastigotes. In a first approach, trypomastigotes were pre-treated for 1 h with lopinavir and nelfinavir at 1, 5 and 10 μM. Only the concentration of 10 µM of both HIV-PIs was able to reduce the interaction with LLC-MK_2_ epithelial cells by around 38%, whilst the adhesion of parasites to RAW murine macrophages decreased 37% and 31%, respectively.^37^ Similarly, both HIV-PIs also decreased the interaction process with LLC-MK_2_ cells when parasites were pre-treated with a lower concentration (1 μM) for longer periods (2 and 4 h). However, regarding the interaction with RAW macrophages, nelfinavir at 1 µM increased the inhibition from 46 to 61% as the time interval of the pre-treatment rose from 2 to 4 h, while the pre-treatment with 1 µM of lopinavir reduced the adhesion of parasites, respectively, in 25 and 48%.^42^

The effects of both HIV-PIs against *T. cruzi* (Y strain) intracellular amastigotes were also assessed on infected LLC-MK_2_ and RAW cells. In a first approach, both infected systems were treated with single doses of either nelfinavir or lopinavir. Regarding the LLC-MK_2_ systems, all tested concentrations of lopinavir were able to dramatically impair the survival of intracellular amastigotes, presenting an IC_50_ value of 5.9 µM after 72 h of drug-contact. On the other hand, nelfinavir was capable of decreasing the number of amastigotes per epithelial cell; however, with the concentrations employed, it was not possible to calculate the IC_50_ value. In infected RAW macrophage systems, a huge prominent effect was seen with both drugs, for which the IC_50_ doses after 48 h of treatment were calculated as 3 µM (nelfinavir) and 4.8 µM (lopinavir).^42^

After that, we speculate whether a daily treatment with both HIV-PIs would be more effective. Therefore, non-cytotoxic concentrations of nelfinavir and lopinavir were employed in a diary treatment protocol. After 72 h of treatment under these conditions, nelfinavir and lopinavir were able to reduce drastically the association indexes with LLC-MK_2_ by around 75% when the maximum concentrations were employed (3.12 µM and 6.25 µM, respectively) (Fig. 3). As a result, low IC_50_ doses were calculated as 1.3 µM for nelfinavir and 2.2 µM for lopinavir. The daily treatment for 48 h of infected RAW macrophages also promoted a similar effect, where 90% of reductions in the association indexes were observed with both compounds at their highest doses. Nelfinavir displayed an IC_50_ value of 1.6 µM and lopinavir 3.2 µM against intracellular amastigotes.^42^

**Fig. 3:**
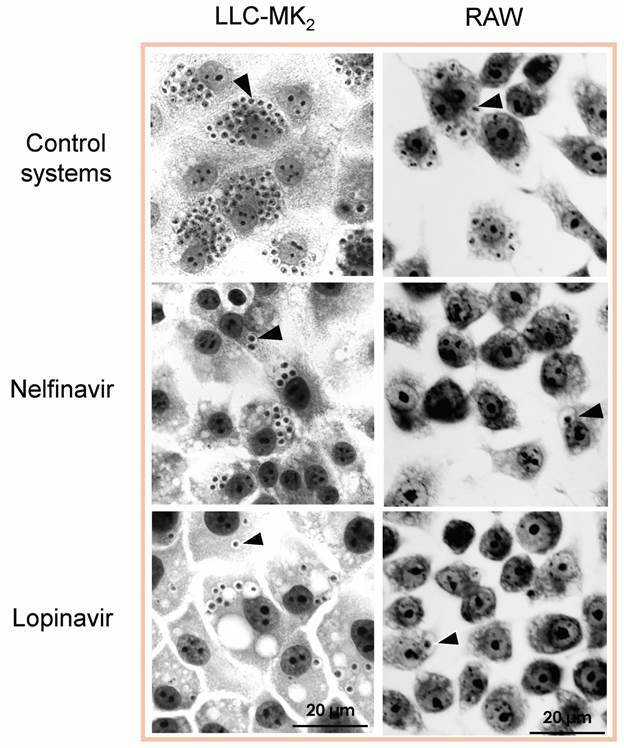
bright field microscopy of the susceptibility of *Trypanosoma cruzi* intracellular amastigotes to the treatment with the human immunodeficiency virus aspartyl peptidase inhibitors (HIV-PIs) nelfinavir and lopinavir in LLC-MK_2_ cells and RAW macrophages. The host cells were previously infected with trypomastigotes for 24 h at 37ºC and then treated daily or not (control) with nelfinavir and lopinavir for 48 h (RAW macrophages) and 72 h (LLC-MK_2_ cells). Finally, the association index was determined by counting 200 cells in each of duplicated coverslips. The arrowheads point to the intracellular parasites. Adapted from Sangenito and coworkers.^37^

In the study of Bellera and co-workers,^34^ the authors infected H9C2 rat myoblasts with trypomastigotes and then treated the systems with saquinavir at 10 µM. After 96 h of drug-contact, the number of intracellular amastigotes per myoblast cell was reduced by 85%. In the same study, a different approach was performed by the authors. Trypomastigotes were first treated with saquinavir at 10 μM and then put to infect H9C2 rat myoblast cells. The systems were then submitted to new doses of the PI at 10 μM for additional 48 h. At the first moment, saquinavir impaired in 90% the cell invasion. Subsequently, the count of internalised parasites was also drastically reduced, indicating that early division of newly formed amastigotes was blocked.^34^

*HIV-PIs present low toxicity for mammalian cells and have good selective indexes* - The HIV-PIs had an important impact on the viability of the clinically relevant forms (i.e., trypomastigotes and amastigotes) of *T. cruzi.* Moreover, it is important to note that these compounds had very low toxicity, as verified against different *T. cruzi* host cells: H9C2 rat myoblasts cells,^34^ LLC-MK_2_ monkey epithelial cells and RAW 264.7 murine macrophages.^42^ Indeed, very good selective indexes were obtained for nelfinavir and lopinavir in the correlation between *T. cruzi* and the mammalian host cells such as RAW and LLC-MK_2_.^35^ ^,^ ^42^ Taking lopinavir as an example, this HIV-PI presented selective indexes up to 37.7 in the relation to trypomastigotes and RAW/LLC-MK_2_ cells. Lopinavir was also 15.6 and 27.4 times more toxic for amastigotes than for RAW and LLC-MK_2_ cells, respectively.^35^ ^,^ ^42^ These results are extremely important especially in case against the intracellular and replicative amastigotes, the main form in terms of maintenance and spreading of the infection in the human body.^2^ Still following the example of lopinavir, the IC_50_ calculated for *T. cruzi* amastigotes in *in vitro* assays was 5.9 µM.^42^ This value represents approximately to 3.65 µg/mL of the drug, a concentration lower than that usually detected in the blood plasma of patients receiving HAART, that ranging from 5.8 to 15.5 µg/mL of lopinavir between each dose interval.^43^ ^,^ ^44^

Perspectives: looking to the future

With all the data presented here, it is clear that HIV-PIs, particularly lopinavir and nelfinavir, have an interesting potential to be repositioned. Both HIV-PIs are able to induce several and crucial physiological changes that directly impact the parasite life cycle. Analysing this impact against the clinically relevant forms of *T. cruzi*, we highlight that HIV-PIs reduce drastically the survival of trypomastigote forms, prevent the process of adhesion/internalisation of these forms with mammalian host cells and affect the proliferation and survival of the intracellular amastigote forms. Therefore, given these observations, our research group aims in a near future to combine these compounds with benznidazole, trying to find the best concentrations that, while being more effective, are also less toxic, starting with *in vitro* and then *in vivo* assay models.

In Conclusion

The lack of investment on neglected tropical diseases associated with the limitation of existing therapeutic options makes drug repositioning an intelligent and necessary strategy. This statement becomes even clearer when we analyse the situation of CD, which has practically only one medication. In view of this scenario, several research centres, independent institutions and also the Drugs for Neglected Diseases *initiative* (DND*i*) focuses on the discovery of alternatives for treating this illness and also improving the current treatment with benznidazole. Indeed, the controlled proof-of-concept phase II clinical trials focused on compare repositioned drugs with benznidazole or in combination with it.^10^ Therefore, given all the information presented here, it is more than reasonable to test the HIV-PIs in combination with benznidazole in order to reduce toxicity and increase efficacy in the treatment of CD.
